# Hormonal Receptor Immunochemistry Heterogeneity and ^18^F-FDG Metabolic Heterogeneity: Preliminary Results of Their Relationship and Prognostic Value in Luminal Non-Metastatic Breast Cancers

**DOI:** 10.3389/fonc.2020.599050

**Published:** 2021-01-12

**Authors:** Nicolas Aide, Nicolas Elie, Cécile Blanc-Fournier, Christelle Levy, Thibault Salomon, Charline Lasnon

**Affiliations:** ^1^ Nuclear Medicine Department, University Hospital, Caen, France; ^2^ INSERM 1086 ANTICIPE, Normandy University, Caen, France; ^3^ Université de Caen Normandie, UNICAEN, SF 4206 ICORE, CMABIO3, Caen, France; ^4^ Pathology Department, François Baclesse Cancer Centre, Caen, France; ^5^ Breast Cancer Unit, François Baclesse Cancer Centre, Caen, France; ^6^ Nuclear Medicine Department, Hospital Centre, Versailles, France; ^7^ Nuclear Medicine Department, François Baclesse Cancer Centre, Caen, France

**Keywords:** breast cancer, steroid receptors, image processing, computer-aided system, radiomics analysis; ^18^F-FDG PET imaging

## Abstract

**Introduction:**

We aimed to investigate whether ^18^F-FDG PET metabolic heterogeneity reflects the heterogeneity of estrogen receptor (ER) and progesterone receptor (PR) expressions within luminal non-metastatic breast tumors and if it could help in identifying patients with worst event-free survival (EFS).

**Materials and methods:**

On 38 PET high-resolution breast bed positions, a single physician drew volumes of interest encompassing the breast tumors to extract SUV_max_, histogram parameters and textural features. High-resolution immunochemistry (IHC) scans were analyzed to extract Haralick parameters and descriptors of the distribution shape. Correlation between IHC and PET parameters were explored using Spearman tests. Variables of interest to predict the EFS status at 8 years (EFS-8y) were sought by means of a random forest classification. EFS-8y analyses were then performed using univariable Kaplan-Meier analyses and Cox regression analysis. When appropriate, Mann-Whitney tests and Spearman correlations were used to explore the relationship between clinical data and tumoral PET heterogeneity variables.

**Results:**

For ER expression, correlations were mainly observed with ^18^F-FDG histogram parameters, whereas for PR expression correlations were mainly observed with gray-level co-occurrence matrix (GLCM) parameters. The strongest correlations were observed between skewness__ER_ and uniformity__HISTO_ (ρ = −0.386, p = 0.017) and correlation__PR_ and entropy__GLCM_ (ρ = 0.540, p = 0.001), respectively. The median follow-up was 6.5 years and the 8y-EFS was 71.0%. Random forest classification found age, clinical stage, SUV_max_, skewness__ER_, kurtosis__ER_, entropy__HISTO_, and uniformity__HISTO_ to be variables of importance to predict the 8y-EFS. Univariable Kaplan-Meier survival analyses showed that skewness__ER_ was a predictor of 8y-EFS (66.7 ± 27.2 versus 19.1 ± 15.2, p = 0.018 with a cut-off value set to 0.163) whereas other IHC and PET parameters were not. On multivariable analysis including age, clinical stage and skewness__ER_, none of the parameters were independent predictors. Indeed, skewness__ER_ was significantly higher in youngest patients (ρ = −0.351, p = 0.031) and in clinical stage III tumors (p = 0.023).

**Conclusion:**

A heterogeneous distribution of ER within the tumor in IHC appeared as an EFS-8y prognosticator in luminal non-metastatic breast cancers. Interestingly, it appeared to be correlated with PET histogram parameters which could therefore become potential non-invasive prognosticator tools, provided these results are confirmed by further larger and prospective studies.

## Introduction

Breast cancer is the most frequently diagnosed cancer in women (16% of all women’s cancers) in all world regions.^1^ Its incidence is rising as a result of longer life expectancy and changes in risk factors. Breast cancer treatment recommendations are based on histological subtype (ER-positive, HER-2 positive, or triple negative tumors), tumor grade, and stage of the disease. More recently, with the development of DNA microarray gene expression analysis, a molecular classification has been proposed and validated (1–3). However, its clinical use is limited, since these techniques are currently expensive as compared to conventional immunohistochemistry (IHC). An attempt to replicate molecular classification using conventional IHC characteristics of the tumor, including ER, PR, HER-2, and Ki67 showed low concordance with gene expressions profile (4, 5). When it comes to breast cancer staging, 2-deoxy-2[18F]-fluoro-D-glucose (^18^F-FDG) PET/CT is a well-established examination for the initial staging of locally advanced breast cancer (6–9), as it displays excellent capabilities for extra-axillary nodal and distance metastases detection. On the contrary, for the local evaluation of primary breast lesion, ^18^F-FDG PET/CT has so far been outperformed by echography and MRI mainly because of its lack of sensitivity (10, 11). However, with the newly growing development of metabolic heterogeneity features in nuclear medicine, the PET community is regaining interest in the value of ^18^F-FDG PET/CT for the non-invasive biological characterization of primary breast tumors. Until now, PET radiomics have always been confronted with the expression of ER, PR, HER2, and Ki67 (12–15) and PET radiomics certainly seem to represent more than just a binary expression of receptors. Meanwhile, improvement in high-resolution scanning of pathological sections and digital imaging analysis is leading to the rise of digital-IHC. Even though it demands further validation and standardization, this technique can provide computation of texture and distribution parameters for hormonal receptors intra-tumoral heterogeneity (16, 17).

The objective of the present study was therefore to investigate (**i**) if PET metabolic heterogeneity features reflect the heterogeneity of ER and PR expression within luminal breast tumors and (**ii**) if PET metabolic heterogeneity features could help in non-invasively identifying patients with the worst event-free survival (EFS).

## Material and Methods

### Study Population

This study is an ancillary study to a previous monocentric and prospective one conducted in our PET unit (18). From April 2009 to June 2012, that study included newly diagnosed and histologically proven breast cancer for which surgery was indicated in first place without neo-adjuvant chemotherapy. It was approved by the Ethics Committee (CPP Nord Ouest III, reference 2009-10) and all patients gave informed and signed consent.

### PET/CT Acquisitions

All ^18^F-FDG PET/CT acquisitions were performed on a Biograph TrueV (Siemens Healthineers) before any treatment. Patients were fasted during at least 6 h. A high-resolution (HR) breast-dedicated bed position (6 min per bed position) was acquired 75 min after the radiopharmaceutical injection. Data were reconstructed using an algorithm with point spread function (PSF) modeling (HD; TrueX, Siemens Healthineers, 3 iterations, and 21 subsets) with no post-filtering and a 512^2^ matrix size leading to voxels of 1.3 × 1.3 × 1.9 mm (19).

### PET-CT Analysis

Injected dose, time between injection and acquisition and capillary glycaemia were recorded to seek EANM recommendations fulfilment (20). A single observer delineated volumes of interest (VOIs) that encompassed the entire breast tumor by using a gradient-based method implemented in MIM software (MIM software, version 5.6.5). When multiple lesions were depicted, only the biggest lesion was considered. VOIs were then saved as DICOM RT structures and loaded in LifeX v5.10 software (21) (www.lifexsoft.org) to extract SUV_max_, histogram parameters and the following TFs:

- Inverse difference, angular second moment, variance, correlation, entropy, dissimilarity from gray-level co-occurrence matrix (GLCM) that considers the arrangements of pairs of voxels
- coarseness, contrast and busyness from neighborhood gray-level different matrix (NGLDM) that corresponds to the difference of gray-level between one voxel and its 26 neighbors in 3 dimensions.

All textural features fulfilled the benchmark of the image biomarkers standardization initiative (22). Absolute resampling using 64 bins between 0 and 32 (corresponding to the maximum SUV units recorded within PET data) was used for all TFs leading to a size of bin 0.5 (23, 24).

### Immunochemistry

Automated immunohistochemistry using a Ventana Bench Mark Ultra was performed on 4-μm-thick paraffin sections of tumor resection with clone SP1 Ventana for ER (pre-diluted) and clone 1E2 Ventana for PR (pre-diluted). The slides were controlled by an experienced pathologist.

### Digital-Immunochemistry Computation

The ScanScope CS microscope slide scanner (Leica Biosystems) was used to digitize whole slide images of histological sections at 20 × (0.5 µm/pixel) and record them as tiled tiff images.

For each image, regions of interest (ROIs) were drawn using the ImageScope software (Leica Biosystems) in order to select only tumor tissues and remove the artifacts. The images were processed as reported in the previous study (25). Briefly, squares of 2000 pixels size corresponding to 1 mm^2^ area were used in this study. The squares were generated to fit the area of the ROI. A ratio between the stained area (brown color) and the surface of tissue was computed and assigned to each square based on their coordinates. Local ratio computed for each square was ranked according to the following ten intervals: level 0 (0–10%), level 1 (>10–20%), level 2 (>20–30%), level 3 (>30–40%), level 4 (>40–50%), level 5 (>50–60%), level 6 (>60–70%), level 7 (>70–80%), level 8 (>80–90%), and level 9 (>90–100%). The ranks then formed the basis for the co-occurrence matrix used to compute Haralick texture parameters. The classical Haralick parameters (26) were computed from the normalized co-occurrence matrix: contrast, homogeneity, dissimilarity, entropy, energy, and correlation. The descriptors of the distribution shape were also computed: skewness and kurtosis.

### Statistical Analysis

**Figure 1:**
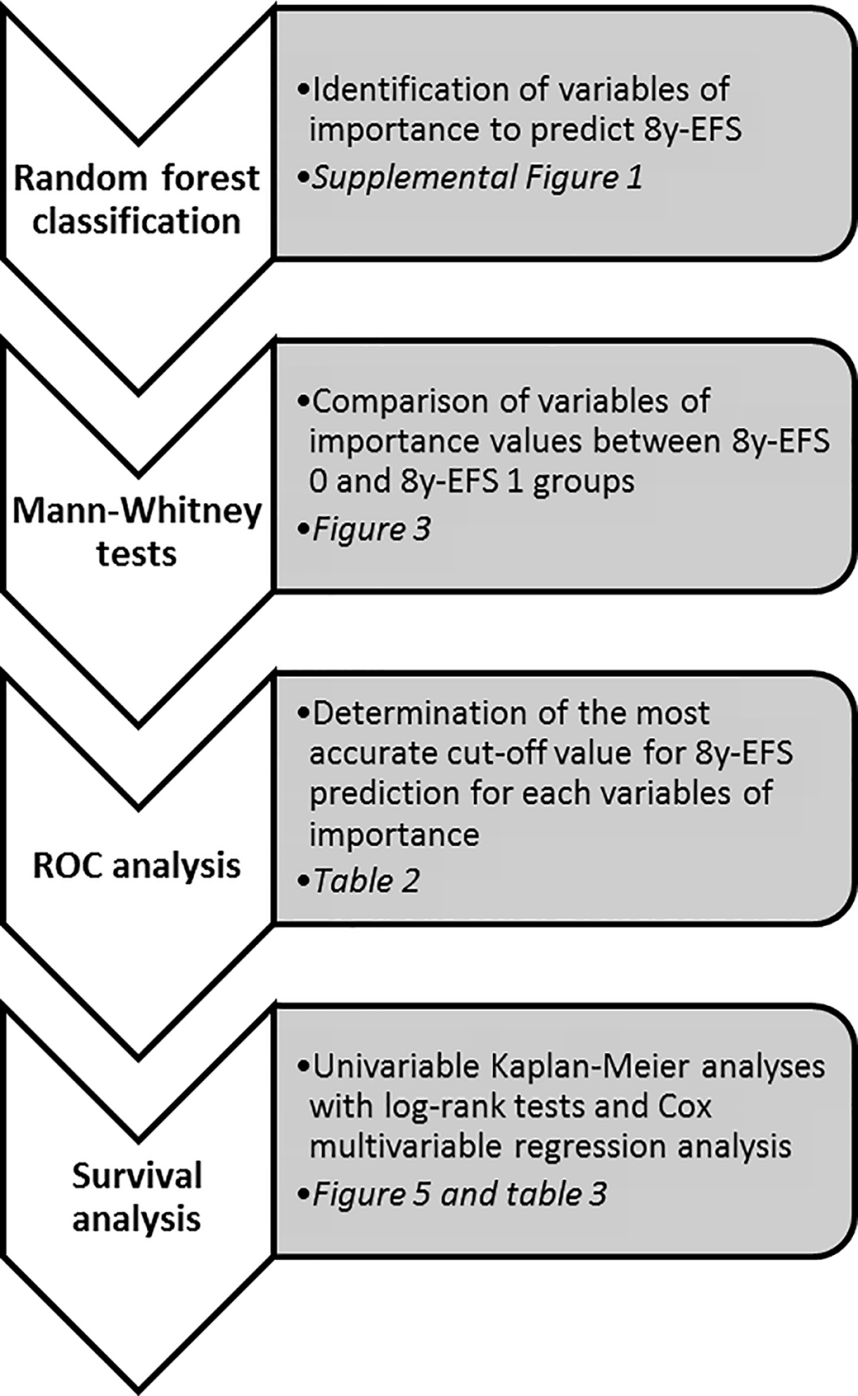
Statistical process summary.

Quantitative data are presented as mean (standard deviation). Correlation between immunochemistry parameters and PET parameters were explored using Spearman correlation tests and matrixes. Variables of interest to predict the occurrence of an event at 8 years (EFS-8y) were sought by means of a random forest classification incorporating the following variables: age, histology, clinical stage, Elston and Ellis grade, molecular subtype classification (27), all immunochemistry parameters and all PET parameters. This analysis implemented classification and regression trees (CART, n = 100) as well as the bootstrapping aggregating (bagging) method previously proposed by Breiman (28–30). For the validation, i.e. the training accuracy, the internal check in RF itself was used, based on the prediction error using the Out-Of-Bag (OOB) estimates of classification error: the smaller the OOB error rate, the better the model is able to classify patients according to their EFS at 8 years (8y-EFS 0 and 8y-EFS 1). The importance of variables in classification was assessed by measuring the mean decrease accuracy (31) of class prediction. Variables of importance were compared between 8y-EFS 0 and 8y-EFS 1 groups using non-parametric Mann-Whitney tests. Receiving operating characteristics (ROC) analyses for 8y-EFS were then undertaken on variables identified as significantly different between groups to define optimal cut-off values based on the Youden index. Eight-year EFS analyses were finally performed using univariable Kaplan-Meier analyses, log-rank tests for comparison of survival curves and finally multivariable Cox regression analysis. The end-point used for survival analysis was the time from diagnosis until relapse or progression, unplanned retreatment, or death as a result of breast cancer. When appropriate, non-parametric Mann-Whitney tests and Spearman correlation tests were used to explore the relationship between clinical data and tumoral heterogeneity variables. Graph and statistical analysis were performed on XLSTAT Software (XLSTAT: Data Analysis and Statistical Solutions for Microsoft Excel. Addinsoft (2017)). For all statistical tests, we retained a two-tailed p value of less than 0.05 as statistically significant. Statistical process is summarized in **Figure 1**.

## Results

### Patients and PET Characteristics

Sixty-three patients were referred for the staging of breast carcinoma from April 2009 to June 2012. Twenty-five patients were excluded from the analysis, leading to a final database of 38 patients. The causes of exclusion were as follows: PET-CT not performed prior to surgery (n = 8), metastatic tumors on initial staging (n = 4), missing data (n = 1), breast lesions not ^18^F-FDG avid (n = 3), hormonal receptors (ER and PR) negative tumors (n = 7), IHC slide unusable (n = 1), and volume of interest too small to be analyzed with LifeX software (n = 1). Patient characteristics are displayed in **Table 1**. Thirty-four tumors were ER+/PR+ and 4 tumors were ER+/PR−. All patients underwent an adjuvant treatment: radiotherapy and hormonotherapy in 10 patients (26.3%) or chemotherapy, radiotherapy and hormonotherapy +/− trastuzumab in case of HER2+ tumors in 28 patients (73.7%). Mean injected dose and uptake time was 4.10 (0.56) MBq/kg and 81.6 (8.4) min, respectively.

**Table 1:** Patients characteristics.

Characteristics	All patients (n = 38)	
**Age (years, mean [min**–**max])**	55 [32–80]	
**Histology (n, %)** Invasive ductal carcinoma	30	78.9
Invasive lobular carcinoma	2	5.3
Tubular carcinoma	1	2.6
Mixed carcinoma	5	13.2
**Tumor stage (n, %)** 1	10	26.3
2	20	52.6
3	8	21.1
**Nodal stage (n, %)** 0	11	28.9
1	17	44.7
2	5	13.2
3	5	13.2
**Elston and Ellis grade (n, %)** I	4	10.5
II	22	57.9
III	12	31.6
**Molecular subtype classification (n, %)** Luminal A	24	63.2
Luminal B/HER-2 negative	10	26.3
Luminal B/HER-2 positive	4	10.5

### Correlations Among Descriptors of the Distribution Shape and Haralick Texture Parameters of Estrogen and Progesterone Receptors Expression

**Figure 2:**
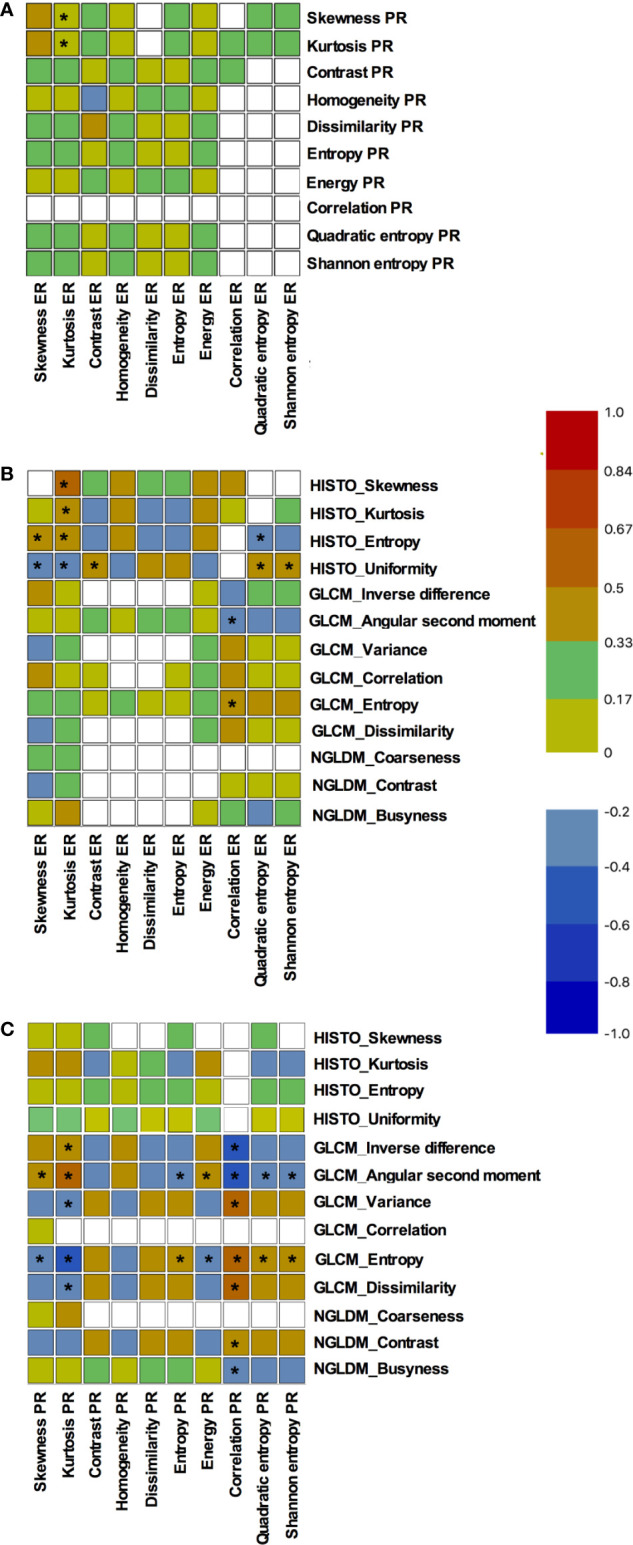
Correlations among distribution descriptors and Haralick texture parameters of estrogen and progesterone receptors expression. Results are presented as Spearman correlations maps: **(A)** correlations between estrogen and progesterone receptors expression parameters, **(B)** correlations between ^18^F-FDG textural parameters and estrogen receptors expression parameters, **(C)** correlations between ^18^F-FDG textural parameters and progesterone receptors expression parameters. The blue color corresponds to a correlation close to −1 and the red color corresponds to a correlation close to 1. The green corresponds to a correlation close to 0. * represents significant correlations (p < 0.05).

Apart from skewness__ER_ that fairly correlated with both skewness__PR_ and kurtosis__PR_ with Spearman coefficients equal to 0.396 and 0.361 (p = 0.015 and p = 0.026), respectively, none of the ER and PR distribution descriptors or Haralick texture parameters were correlated to each other (**Figure 2A**).

### Relation Between ^18^F-FDG Textural Parameters and Intra-Tumoral Estrogen Receptors Expression

Relationship between variables can be seen in **Figure 2B**. Correlations were mainly observed with ^18^F-FDG histogram parameters. Indeed, all PET histogram parameters were fairly correlated to kurtosis__ER_ with Spearman coefficients ranging from −0.338 to 0.410. Moreover, uniformity__HISTO_ was significantly but fairly correlated to skewness__ER_, contrast__ER_, quadratic entropy__ER_ and shannon entropy__ER_ (ρ = −0.386, p = 0.017; ρ = 0.329, p = 0.044; ρ = 0.361, p = 0.027, and ρ = 0.333, p = 0.042, respectively). Finally, entropy__HISTO_ was also fairly correlated to skewness__ER_ and quadratic entropy__ER_ (ρ = 0.369, p = 0.023; ρ = −0.344, p = 0.035, respectively).

When considering GLCM PET parameters, we observed correlations only between correlation__ER_ and both angular second moment__GCLM_ and entropy__GLCM_. Overall the PET parameter displaying the more numerous statistically significant correlations (n = 5) with intra-tumoral estrogen receptors expression was uniformity__HISTO_ with the strongest correlation being observed with skewness__ER_: ρ = −0.386, p = 0.017.

### Relation Between ^18^F-FDG Textural Parameters and Intra-Tumoral Progesterone Receptors Expression

Relationship between variables can be seen in **Figure 2C**. None of histogram PET parameters were correlated to intra-tumoral progesterone receptors expression parameters. Correlation__PR_ was the parameter displaying the maximal rate of statistically significant correlations with PET parameters (n = 7). It was fairly correlated to inverse difference__GLCM_, angular second moment__GLCM_, variance__GLCM_, entropy__GLCM_, dissimilarity__GLCM_, contrast__NGLDM_ and busyness__NGLDM_ (ρ = −0.449, p = 0.005; ρ = −0.525, p = 0.001; ρ = 0.469, p = 0.003; ρ = 0.540, p = 0.001; ρ = 0.456, p = 0.004; ρ = 0.398, p = 0.014; ρ = −0.322, p = 0.049).

Angular second moment__GLCM_ and entropy__GLCM_ were the PET parameters displaying the more numerous statistically significant correlations with intra-tumoral progesterone receptors expression. They both correlated to all IHC parameters, with the exception of contrast__PR_, homogeneity__PR_, and dissimilarity__PR_. The strongest correlation was observed between entropy__GLCM_ and correlation__PR_: ρ = 0.540, p = 0.001.

### Survival Data Analysis

**Figure 3:**
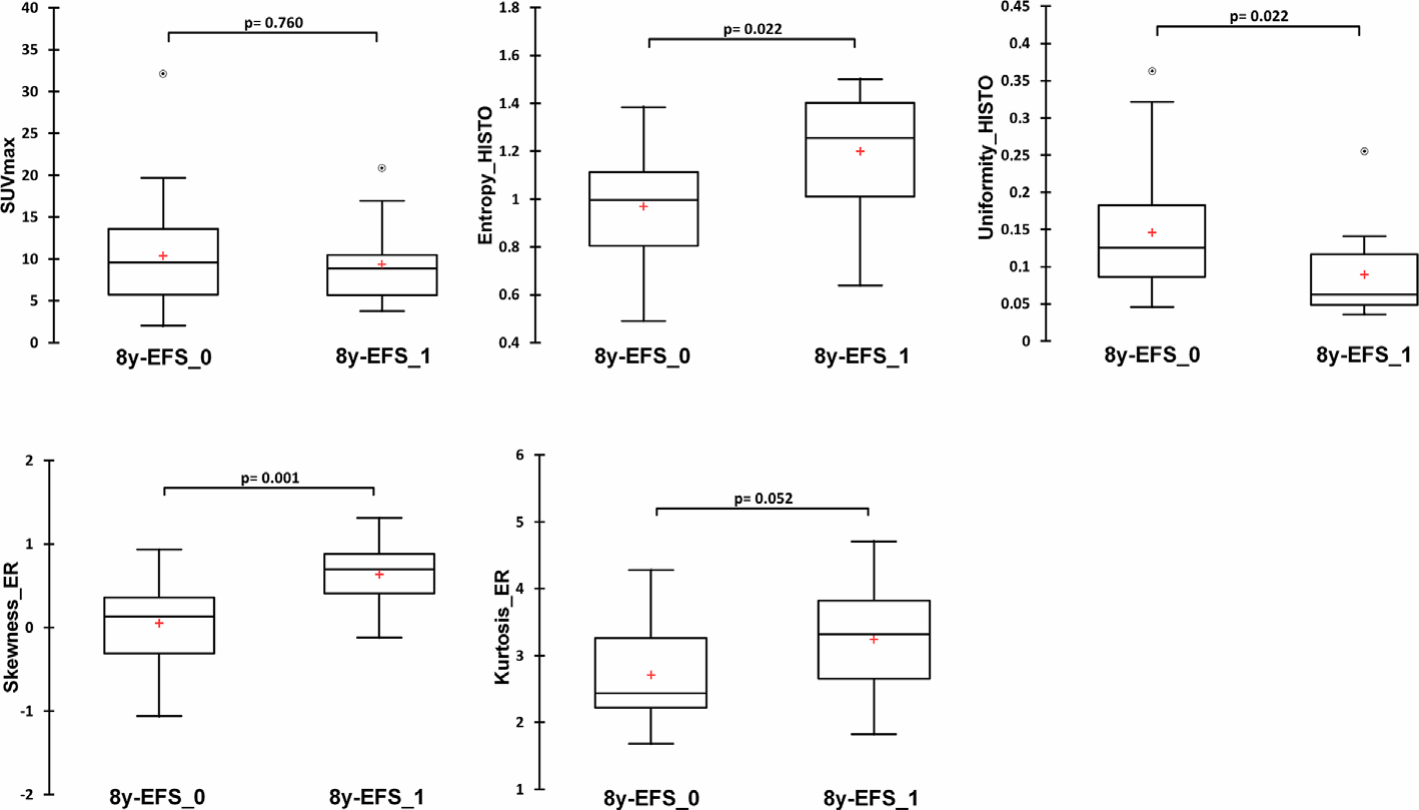
Comparison of immunochemistry and PET variables of importance identified by random forest analysis between 8y-EFS_0 and 8y-EFS_1 patients (SUVmax, entropy__HISTO_, uniformity__HISTO_, skewness__ER_, and kurtosis__ER_). Data are shown as Tukey boxplots with (○) representing outliers.

**Figure 4:**
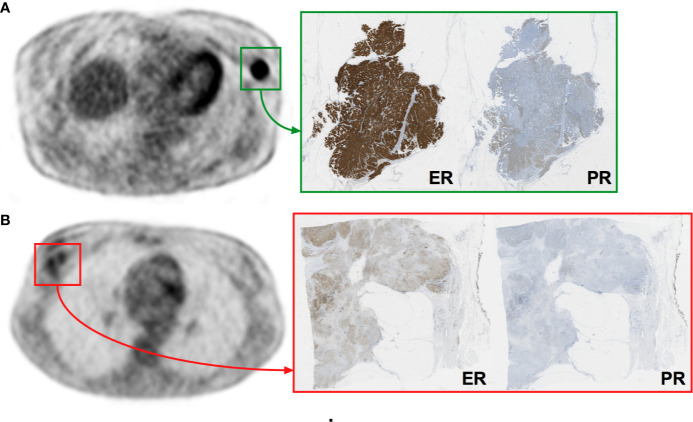
Representative images of PET and digital-immunochemistry images. Patient **(A)** was a 74-year-old women with a luminal ER+/PR+ tumor staged II presenting homogeneous IHC and PET characteristics (skewness__ER_ = −1.06, entropy__HISTO_ = 0.66, uniformity__HISTO_ = 0.24) who experienced no event at 8 years (8y-EFS_0). Patient **(B)** was a 34-year-old women with a luminal ER+/PR+ tumor staged III presenting heterogeneous IHC and PET characteristics (skewness__ER_ = 1.31, entropy__HISTO_ = 1.50, uniformity__HISTO_ = 0.04) who experienced an event at 8 years (8y-EFS_1).

**Figure 5:**
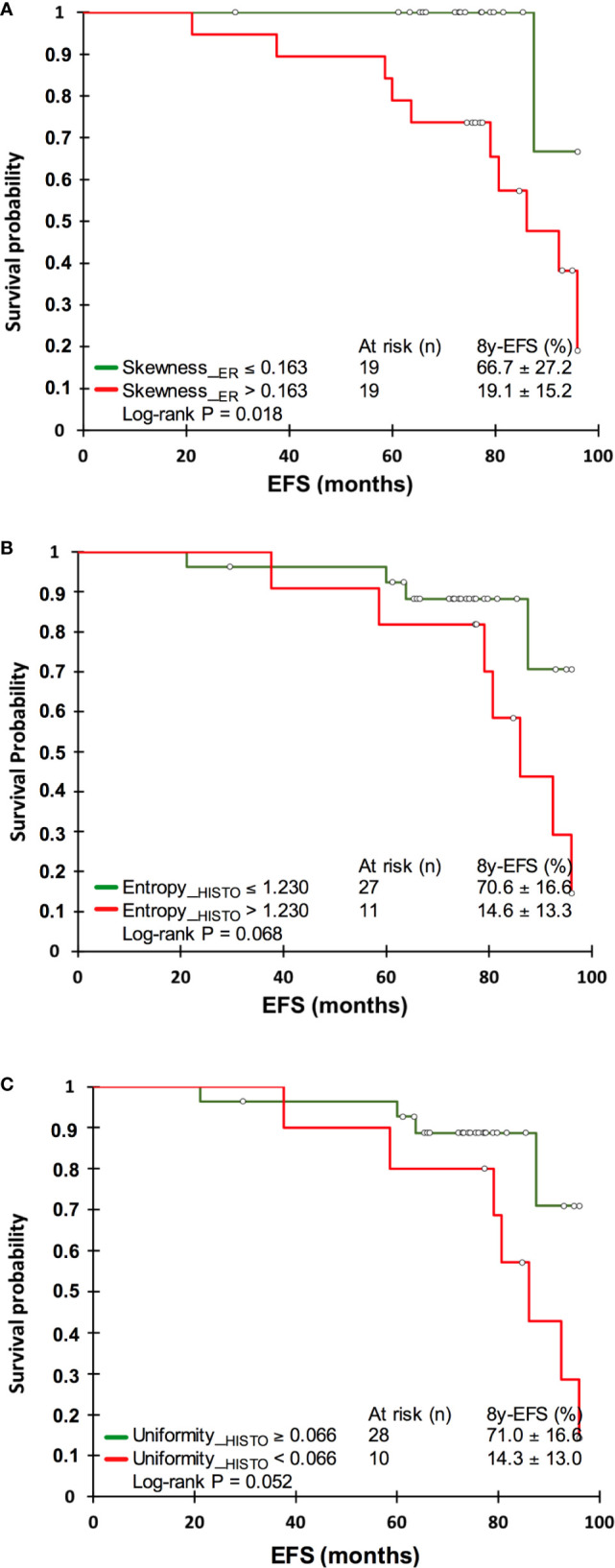
Kaplan-Meyer analyses for skewness__ER_ **(A)**, entropy__HISTO_ **(B)**, and uniformity__HISTO_ **(C)**.

**Figure 6:**
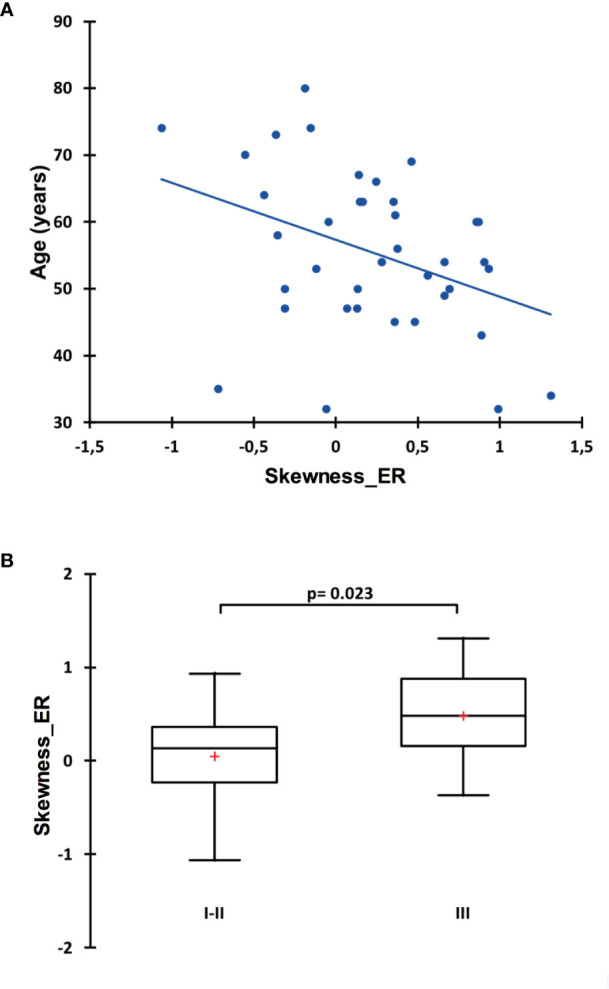
Spearman correlation between skewness__ER_ and the age at diagnosis **(A)** and comparison of skewness__ER_ between clinical staged I–II versus staged III patients. Data is shown as Tukey boxplots **(B)**.

The statistical process for this specific part is summarized in **Figure 1**. The median follow-up was 6.5 years (range: 2.5–9.1 years) and with 11 recorded events, the 8y-EFS was 71.0% in the entire population. Among the 11 recorded events, 8 were metastatic recurrences, 2 were contralateral recurrences, and 1 was a local recurrence. The median time to recurrence from the date of diagnosis was 78 months ranging from 21 to 96 months. Of note, 4 deaths were recorded over the 8-year follow-up. Random forest classification found age, clinical stage, SUV_max_, skewness__ER_, kurtosis__ER_, entropy__HISTO_, and uniformity__HISTO_ to be variables of importance to predict the 8y-EFS (**Supplemental Figure 1**). The OOB estimate was equal to 28.9%. Mean skewness__ER_ and mean entropy__HISTO_ were significantly higher (p = 0.001 and p = 0.022, respectively), whereas mean uniformity__HISTO_ was significantly lower (p = 0.022) in 8y-EFS_1 patients (**Figure 3**). There were no significant difference in SUV_max_ and kurtosis__ER_ values between 8y-EFS_0 and 8y-EFS_1 patients (p = 0.760 and p = 0.052, respectively). Representative images of PET and digital-immunochemistry images are displayed in **Figure 4**. On ROC analyses, optimal cut-off values for skewness__ER,_ entropy__HISTO_ and uniformity__HISTO_ to predict 8y-EFS were equal to 0.163, 1.23, and 0.066, respectively (**Table 2**). Univariable Kaplan-Meier survival analyses found that skewness__ER_ was a predictor of 8y-EFS whereas entropy__HISTO_ and uniformity__HISTO_ were not, although statistical significance was almost reached (**Figure 5**). On multivariable analysis including skewness__ER_ and other well-known prognosticators [age, clinical stage (I–II versus III)], all the statistics for the test of the null hypothesis are significant and we can conclude that considering explanatory variables provides significant additional information. There was no violation of the proportional hazards assumption. However, regression coefficients showed that none of the parameters were independent predictors of 8y-EFS (**Table 3**). Indeed, we found a significant negative correlation between skewness__ER_ and age (ρ = −0.351, p = 0.031) with skewness__ER_ values higher in youngest patients (**Figure 6A**). Moreover, skewness__ER_ was significantly higher in clinical stage III tumors (p = 0.023, **Figure 6B**). Of note, ER expression was scored + in 2 patients (5.3%), ++ in 6 patients (15.8%), and +++ in 30 patients (78.9%) by IHC analysis. Skewness__ER_ was not significantly different between patients scored +, ++, or +++ (p = 0.508, **Supplemental Figure 2**). A quantification of ER expression in percentage was also available for 35 patients with a mean value equal to 88.5% (± 15.5). It was not significantly correlated with skewness__ER_ (p = 0.207, ρ = 0.048).

**Table 2:** ROC analyses for 8-year event free survival for skewness__ER_, entropy__HISTO_, and uniformity__HISTO_.

Variable	AUC	Standard error	Lower bound (95%)	Upper bound (95%)	P	Cut-off value
Skewness__ER_	0.828	0.083	0.666	0.991	<0.0001	>0.163
Entropy__HISTO_	0.737	0.113	0.515	0.960	0.036	>1.230
Uniformity__HISTO_	0.741	0.116	0.514	0.968	0.038	<0.066

AUC, area under the curve.

**Table 3:** Cox regression analysis.

Test of the null hypothesis								
Statistic	DF	Chi-square	P				
Likelihood ratio test	3	10.60	0.014				
Score test	3	10.86	0.012				
Wald test	3	9.24	0.026				
**Regression coefficients**							
**Variable**	**Value**	**Standard error**	**Wald Chi-square**	**P**	**HR**	**HR lower bound (95%)**	**HR upper bound (95%)**
Skewness__ER_	0.860	0.834	1.063	0.303	2.363	0.461	12.119
Age	−0.033	0.033	1.406	0.306	0.967	0.907	1.031
Clinical stage III	1.245	0.858	2.105	0.147	3.474	0.646	18.681

HR, Hazard ratio.

## Discussion

The first and interesting finding of the present study is the quasi-absence of correlation between ER and PR descriptors of the distribution shape and Haralick texture parameters. This seems to indicate that their heterogeneity expressions are independent and could have different meanings and clinical consequences. Here, we decided to focus on EFS and it appeared that immunochemistry histogram parameters of estrogen receptors, and especially skewness, are predictors of 8y-EFS together with age and clinical stage, whereas none of the progesterone receptors were. Moreover, correlations of ER and PR parameters with PET histogram and textural parameters were clearly different. The ER immunochemistry heterogeneity was mainly correlated to PET histogram parameters, whereas PR immunochemistry heterogeneity was mainly correlated to second-order GLCM-derived PET textural features. Interestingly, skewness__ER_ was a significant predictor of 8y-EFS but not an independent one. Indeed, it was related to both the age of the patient at diagnosis and the clinical stage of the disease: estrogen receptors heterogeneity was higher in youngest patients and in higher-staged diseases. We can hypothesize that ER heterogeneity could be linked to more aggressive tumors. Returning to the PET methodology, the use of a HR PET acquisition to compute ^18^F-FDG heterogeneity parameters (PSF algorithm and 1.3 × 1.3 × 1.9 mm voxels) is a strength. Indeed, it has been previously shown that the type of reconstruction as well as the voxel size, are important considerations when computing ^18^F-FDG heterogeneity (19) especially in small lesions like those bearing breast cancer. However, even though high-resoluted histograms of PET parameters were significantly but fairly correlated to ER immunochemistry ones (especially skewness__ER_, kurtosis__ER_, entropy__HISTO_ and uniformity__HISTO_), PET parameters appeared to be less discriminant for 8y-EFS than immunochemistry ones. Nevertheless, we can notice that log-rank tests for entropy__HISTO_ and uniformity__HISTO_ almost reached statistical significance and that a larger study could have displayed more discriminant results.

Previously in the study of Antunovic et al. (13), using PET metabolic heterogeneity features, two clusters were obtained by the unsupervised hierarchical clustering analyses with different imaging signatures. Besides, these signatures were significantly associated with different molecular subtypes. Ha et al. (14) also performed an unsupervised tumor clustering using a radiomics pattern which resulted in 3 tumor clusters. The expression of histopathological factors between their clusters was different for Ki67. Of note, one cluster displayed higher estrogen and progesterone receptors (ER and PR) expression, but statistical significance was not reached. Lemarignier et al. (15) found a trend for lower local heterogeneity in hormone-positive breast cancer even though statistical significance was no longer observed after correction for multiple testing. Thus, all these results together with ours are first-evidences of a complementary role of imaging features, together with standard PET metrics for a clinically relevant *in vivo* characterization of breast cancer that could lead to a personalization of therapeutic management. The perspectives would be (i) to assess the clinical impact of these results, in particular by offering patients deemed to be at risk of recurrence a closer post-therapeutic monitoring and (ii) to test other innovative tracers such as ^18^F-Fluoroestradiol. Data from a meta-analysis evaluating the ability of ^18^F-Fluoroestradiol for the determination of tumor ER status (32) suggested acceptable diagnostic performance of this radiopharmaceutical despite a weakness in terms of sensitivity [pooled sensitivity = 82% (95% CI: 74–88%), pooled specificity = 95% (95% CI: 86–99%)]. However, to date, there is no data clearly documenting the clinical consequences of patient management following diagnosis with ^18^F-Fluoroestradiol PET. Documenting the intra-tumoral heterogeneity of estrogen receptors using this tracer has not yet been investigated and could be of interest.

It is worth noticing that our findings, even if innovative, were observed in a small cohort and have to be validated by a larger clinical study. The lack of statistical significance might also be due to the limited spatial resolution of an analogic system and it could be wise to test innovative digital systems in future projects. Of note, PET third-order textural features were not considered in the present study because their computation was very far from that used for immunochemistry parameters. Indeed, immunochemistry parameters could only use histograms or co-occurrence matrixes. Also, inter-observer variability for the quantification of metabolic heterogeneity was not presently assessed. However, we have taken care to choose one of the most reproducible delineation methods, namely, a gradient-based method (33), thus limiting the variability linked to the operator. However, other sources of variability must be taken into account regarding the clinical export of such results: software, PET systems, reconstructions, etc. Therefore, we acknowledge that harmonization strategies will be necessary anyway. Finally concerning immunochemistry methodology, the age of the samples jeopardized the achievement of Ki67 expression heterogeneity exploration because of faint immunostaining, not enabling the digital-immunochemistry computation. For HER2 status, international standards require that it be tested at the time of diagnosis, therefore on biopsies. The recommendations say that it is not necessary to repeat it systematically on the piece of excision, because there is a good agreement between the HER2 status tested on the biopsy and remade on the piece, due to a usually homogeneous distribution when expressed (34–36).

To conclude, a heterogeneous distribution of estrogen receptors within the tumor in immunochemistry appeared as an event-free prognosticator in luminal non-metastatic breast cancers. Furthermore, estrogen receptors heterogeneity is higher in youngest patients and the highest-graded tumors. Interestingly, this appeared to be correlated with PET histogram parameters which could therefore become potential tools to reflect the tumor estrogen receptors heterogeneity, provided these results are confirmed by further larger and prospective studies.

## Data Availability Statement

The raw data supporting the conclusions of this article will be made available by the authors, upon reasonable request.

## Ethics Statement

The studies involving human participants were reviewed and approved by CPP Nord Ouest III, reference 2009-10. The patients/participants provided their written informed consent to participate in this study.

## Author Contributions

Study conception and design: NA and CLa. Screening and inclusion of patients: CLe. Data collection: TS and CLa. PET/CT analysis: TS. Immunochemistry: CB-F. Digital-immunochemistry computation: NE. Statistical analysis: CLa. Manuscript editing and reviewing: CLa, CB-F, NE, NA, and CLe. All authors contributed to the article and approved the submitted version.

## Funding

This project was funded by an internal call for tenders from the Center François Baclesse.

## Conflict of Interest

The authors declare that the research was conducted in the absence of any commercial or financial relationships that could be construed as a potential conflict of interest.

## Footnotes

1
https://www.who.int/topics/cancer/breastcancer/fr/index1.html.

## Supplementary Material

Supplementary Figure 1 Random forest variables of importance analysis displayed as mean decrease accuracy.

Supplementary Figure 2 Comparison of skewness__ER_ by estrogen receptor expression score (+, ++ or +++). Data is shown as Tukey boxplots.

## Abbreviations

PET, Positron Emission Tomography; ER, Estrogen Receptor; PR, Progesterone Receptor; SUV, Standardized Uptake Value; IHC, Immunohistochemistry; EFS, Event Free Survival; RF, Random Forest Classification; GLCM, Gray-Level Co-occurrence Matrix; ^18^F-FDG, ^18^Fluorodeoxyglucose; HER-2, Human Epidermal Growth Factor Receptor-2; HR, High Resolution; VOI, Volume of Interest; NGLDM, Neighborhood Gray-Level Different Matrix; TF, Textural Feature; ROI, Region Of Interest; SD, Standard Deviation; CART, Classification And Regression Trees; OOB, Out-Of-Bag; ROC, Receiver Operating characteristic Curve; CT, Computed Tomography.
