# Supplementary figures and images for: Hormonal Receptor Immunochemistry Heterogeneity and 18F-FDG Metabolic Heterogeneity: Preliminary Results of Their Relationship and Prognostic Value in Luminal Non-Metastatic Breast Cancers
Source: Front Oncol. 2021 Jan 12;10:599050. doi: 10.3389/fonc.2020.599050 (PMC7837029; doi:10.3389/fonc.2020.599050)

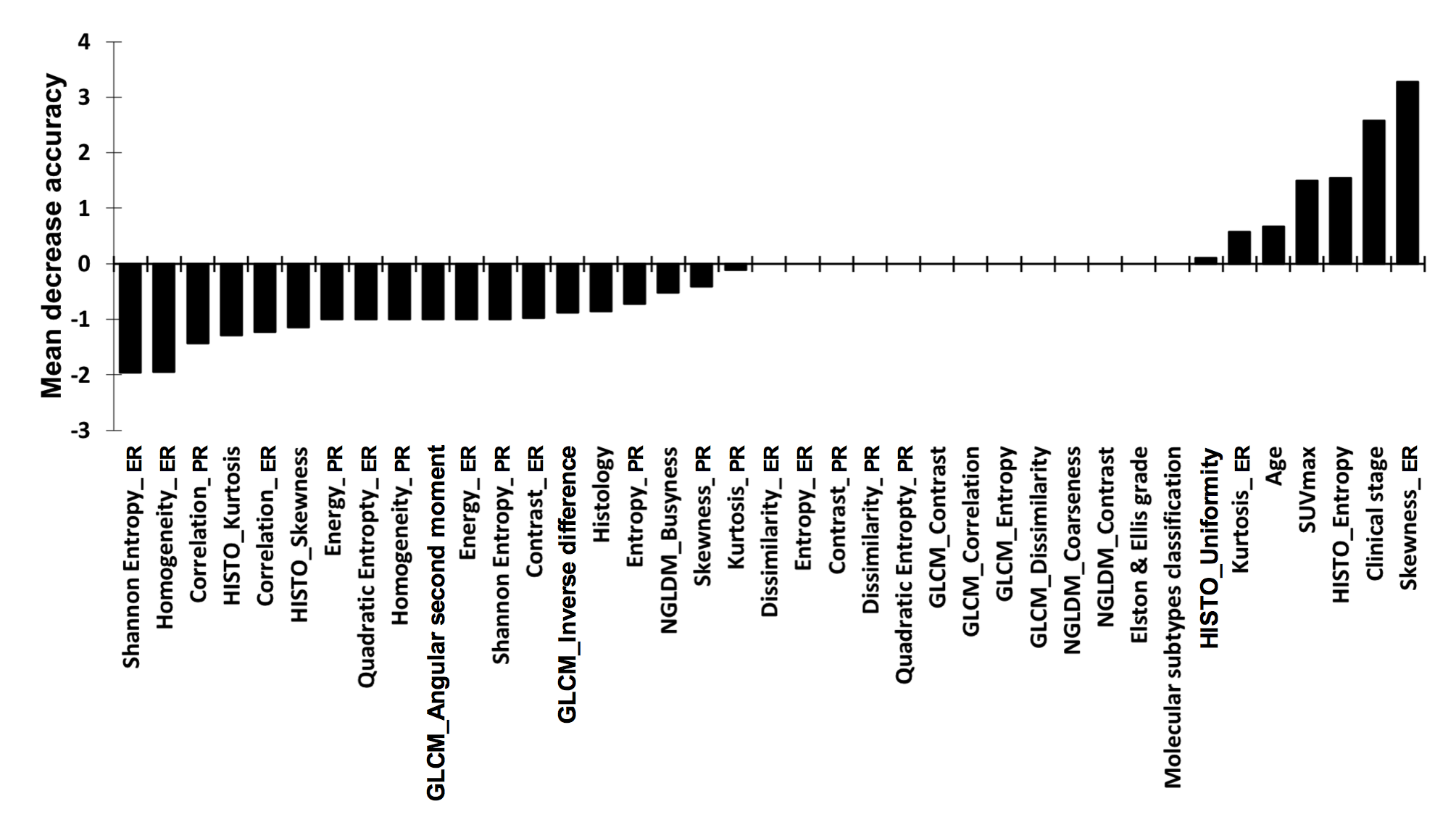

Supplement: Supplementary Figure 1 — Random forest variables of importance analysis displayed as mean decrease accuracy. [file Image_1.tiff]

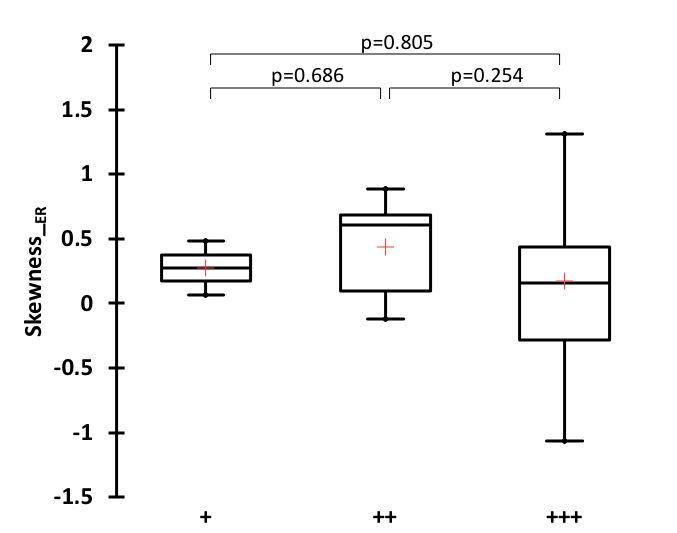

Supplement: Supplementary Figure 2 — Comparison of skewness_ER by estrogen receptor expression score (+, ++ or +++). Data is shown as Tukey boxplots. [file Image_2.png]
